# Linking the Warburg effect to endometrial receptivity: metabolic parallels in embryo implantation

**DOI:** 10.3389/fcell.2025.1683790

**Published:** 2025-11-17

**Authors:** Xiaoyang Zhang, Qingwen Zhu, Weiduo Nie, Xiaoxue Yan, Zhihua Yuan, Leiyu Tian

**Affiliations:** 1 School of Traditional Chinese Medicine, Beijing University of Chinese Medicine, Beijing, China; 2 Dongfang Hospital, Beijing University of Chinese Medicine, Beijing, China; 3 Department of Oncology, Dongzhimen Hospital, Beijing University of Chinese Medicine, Beijing, China

**Keywords:** Warburg effect, endometrial receptivity, embryo implantation, infertility, glycolysis

## Abstract

**Introduction:**

Endometrial receptivity (ER), critical for successful embryo implantation and a major limiting factor in infertility affecting ∼1 in 6 couples globally, remains poorly understood, with few effective interventions targeting the embryo-endometrium interaction. Intriguingly, similarities exist between the implantation microenvironment and the Warburg effect, a metabolic hallmark of cancer characterized by aerobic glycolysis, lactate production, and low pH.

**Methods:**

We conducted a comprehensive review (PubMed search up to April 2025) using keywords related to the Warburg effect (aerobic glycolysis, lactate, mitophagy), infertility (IVF, embryo implantation, TCM), cancer, cytokines (IL-1, LIF, TGF-β), and hormones (estrogen, progesterone).

**Results:**

The review identified significant mechanistic parallels: 1) Blastocysts and trophoblasts establish a pro-receptive, high-lactate/low-pH microenvironment *via* Warburg-like glycolysis; 2) Shared immune modulation occurs (e.g., PI3K-AKT-FOXO1 pathway), balancing inflammatory attachment and immune tolerance; 3) Glycolysis regulates key ER-associated genes (e.g., MRAP2, BCL2L15) and cytokines (IL-1, LIF, TGF-β); 4) Invasive trophoblast behavior mirrors cancer cell invasion, potentially fueled by Warburg metabolism; 5) Hormones (estrogen, progesterone) critically orchestrate glycolytic enzyme expression (e.g., GLUT1, PFKFB3), substrate availability, and lactate-mediated immune suppression to establish this metabolic state.

**Discussion:**

While direct experimental evidence linking the Warburg effect to ER is currently limited, the compelling mechanistic overlap offers a novel paradigm for understanding implantation failure. Targeting this shared metabolic-immune-hormonal axis holds immense potential for developing innovative strategies (e.g., metabolic modulators, refined TCM approaches) to improve ER, enhance embryo implantation rates in infertility (including IVF) and recurrent miscarriage, ultimately advancing global reproductive health. Further research is needed to validate core mechanisms.

## Graphical Abstract

**Figure:**
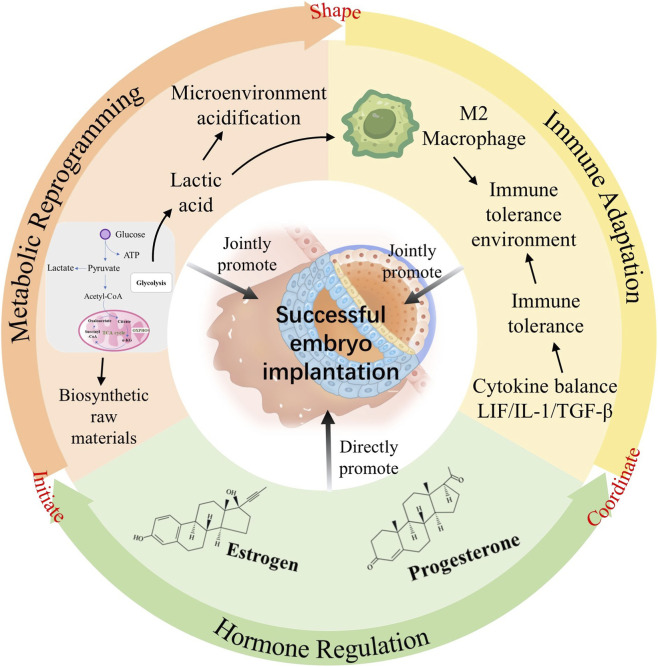

## 1 Background

Infertility is a reproductive challenge currently faced by both Eastern and Western societies. Statistics indicate that approximately one in six couples may experience fertility issues (Bueno-Sánchez et al., 2024). As research progresses and the technologies advance nowadays, investigators have found that the successful implantation of embryos into the endometrium is crucial for achieving and sustaining pregnancy. This process is closely linked to “endometrial receptivity” (Miravet-Valenciano et al., 2015). Endometrial receptivity refers to the uterus’s capacity to receive and accommodate embryos during the 6–10 days following ovulation. The synchrony between high-quality fertilized oocytes and the endometrium is critical for successful conception and pregnancy maintenance, highlighting embryo invasiveness and endometrial receptivity as decisive factors for successful implantation (Lessey and Young, 2019). Investigations indicate that in natural cycles, around 30% of pregnancies fail before embryo implantation (Simón et al., 1998), underscoring that many natural conceptions fail to initiate or complete implantation to achieve ongoing pregnancy (Macklon et al., 2002). Even with assisted reproductive technologies, embryo implantation remains a pivotal determinant of success rates.

Modern clinical approaches primarily aim to improve endometrial receptivity through various interventions to enhance embryo implantation rates and maintain pregnancy. In terms of pharmacological treatment, hormone replacement therapy (such as estrogen and progesterone) is widely used to regulate the endometrial cycle (Paulson, 2019), while traditional Chinese medicine (TCM) (Xin et al., 2018) and Chinese patent medicines (Paulson, 2019; Pan et al., 2023) are also commonly applied.

In TCM research focused on improving endometrial receptivity, several active components have shown potential therapeutic value. Paeoniflorin, the main active compound in Paeonia lactiflora, significantly improves embryo implantation rates in an RU486-induced implantation failure mouse model, and enhances the adhesion ability of human trophoblasts and endometrial cells by upregulating leukemia inhibitory factor (LIF) expression. The mechanism of this effect may be related to the regulation of the LIF signaling pathway (Park et al., 2021). Ginsenosides from Panax ginseng exert their effects through multiple pathways: Rg3 inhibits the VEGFR-2-mediated PI3K/Akt/mTOR signaling pathway, reducing ectopic endometrial angiogenesis and inducing cell apoptosis (Cao et al., 2017); Rg1 alleviates endometrial fibrosis by interfering with the ROS/NLRP3 inflammasome signaling pathway (Song et al., 2023); and Rh3 combats oxidative damage to endometrial cells by activating the Nrf2 signaling pathway (Wang XM. et al., 2020). In compound studies, the PRP formula composed of Paeonia lactiflora, Rehmannia glutinosa, and Perilla frutescens, treated by polysaccharide removal, showed enhanced endometrial receptivity (Eun-Yeong et al., 2019), while the Bushen Cuyun Recipe can improve receptivity by mitigating RU486-induced endometrial damage (Jiang et al., 2023). WSYXD regulates PI3K, HIF-1α signaling, and VEGF expression to assist in the recovery of endometrial receptivity (Xin et al., 2018), promoting endometrial angiogenesis. KLP administration increases endometrial thickness and the number of endometrial glands and pinopodes. In the endometrium, KLP supplementation upregulates the expression of estrogen receptor α, progesterone receptor, endothelial nitric oxide synthase, and integrin αVβ3 in mouse uteri (Pan et al., 2023). These studies provide a robust theoretical foundation for the use of TCM in treating endometrial receptivity disorders.

In assisted reproductive technology, embryo selection (such as PGT-A) and endometrial synchronization are critical steps; however, even with the transplantation of chromosomally normal blastocysts, the ongoing pregnancy rate remains around 50%, highlighting the importance of endometrial receptivity. Other auxiliary methods include acupuncture (Tian et al., 2024; Zhong et al., 2019; Shuai et al., 2015) (possibly through the regulation of uterine blood flow), immunomodulatory treatments (Seles et al., 2024) (targeting Th1/Th2 balance), and emerging intrauterine platelet-rich plasma (PRP) infusion (Li et al., 2023) (possibly promoting endometrial repair *via* growth factors). It is noteworthy that mechanical endometrial injury (scratching) has been proposed to enhance receptivity, but its clinical benefits remain controversial (Santos-Ribeiro et al., 2017). Currently, tools for endometrial receptivity testing (such as RNA-Seq-based ERT) have been developed to more accurately define individualized implantation windows through transcriptomic analysis (He et al., 2021; Ben Rafael, 2021). However, existing treatments mainly focus on the endometrial microenvironment rather than directly intervening in the “embryo-endometrium dialogue.” Future research needs to further elucidate the roles of pathways such as BMP/ACVR2A7 (Monsivais et al., 2021), LIF/STAT38 (Liang et al., 2021), and HOXA109 (Mishra and Modi, 2025) in the establishment of receptivity, to develop more precise intervention strategies.

Research suggests that the high lactic acid and low pH environment created by blastocysts can improve endometrial receptivity (Gurner et al., 2022). This specific microenvironment is achieved through elevated glucose flux facilitated by aerobic glycolysis, supporting heightened biosynthesis in rapidly proliferating cells such as blastocysts, trophoblast cells, and cancers (Gou and Zhang, 2023). This phenomenon, which is widely acknowledged as the “Warburg effect,” is utilized by cancers to support their aggressive growth (Liberti and Locasale, 2016a). Given the functional similarities between blastocyst invasion of the endometrium and cancer invasion of surrounding tissues, it is plausible that cancers employ similar cellular processes and signaling pathways as blastocysts.

Based on this hypothesis, this study conducted a literature review using the keywords “endometrial receptivity,” “Warburg effect,” and “endometrial receptivity and Warburg effect” to explore their correlation, aiming to provide novel insights for clinical management of infertility.

## 2 The concept and process of the Warburg effect

Cells in various tissues of the human body can take up glucose from the blood. Under conditions of sufficient oxygen supply, glucose undergoes complete oxidation, primarily *via* glycolysis in non-proliferative tissues. In this pathway, glucose is metabolized to pyruvate, which enters the mitochondria and is completely oxidized to CO2 and H2O, at the same time releasing substantial energy to drive ATP synthesis. However, when oxygen supply is limited, glucose in the cytoplasm proceeds through glycolysis to generate pyruvate, which is then converted to lactate. This process yields noticeably less ATP per glucose molecule compared to oxidative phosphorylation under aerobic conditions. Lactate production helps regenerate NAD+ to sustain glycolysis, but each glucose molecule produces approximately 28–30 fewer ATP molecules than through oxidative phosphorylation (Berg et al., 2002).

In the 1920s, Otto Warburg observed that tumor cells produce large amounts of lactate even in Adequate oxygen supply (Warburg and Negelein, 1927). Most tumor cells primarily generate ATP through anaerobic glycolysis, leading to lactic acid accumulation, a phenomenon known as the “Warburg effect”. This phenomenon is not limited to tumor cells but also occurs under physiological conditions such as during neural crest migration (Bhattacharya et al., 2020), lymphocyte proliferation (Wang et al., 1976), and macrophage proliferation (Hard, 1970). The “Warburg effect” in tumor cells forms the basis of FDG-PET imaging for tumors and is now widely used in clinical practice (Gallamini et al., 2014).

Why do tumor cells and some proliferating cells alternative a less efficient metabolic mode even when the supply is sufficient? While Warburg and others initially attributed this phenomenon to mitochondrial defects impairing the respiratory chain (WARBURG O, 1956), recent research has shown that mitochondria in tumor cells are functional dynamic and operational (Xu et al., 2015). Current understanding suggests that this metabolic preference may be driven by oncogenic signaling pathways involving kinases and transcription factors (Hsu and Sabatini, 2008; Kroemer and Pouyssegur, 2008). Studies have also demonstrated that estrogen-related receptor (ERR), acting as a coactivator of hypoxia-inducible factor (HIF), interacts with HIF to enhance the expression of glycolytic genes under hypoxic conditions (Ao et al., 2008).

## 3 Embryo implantation and the Warburg effect

### 3.1 Embryo implantation process

**FIGURE 1:**
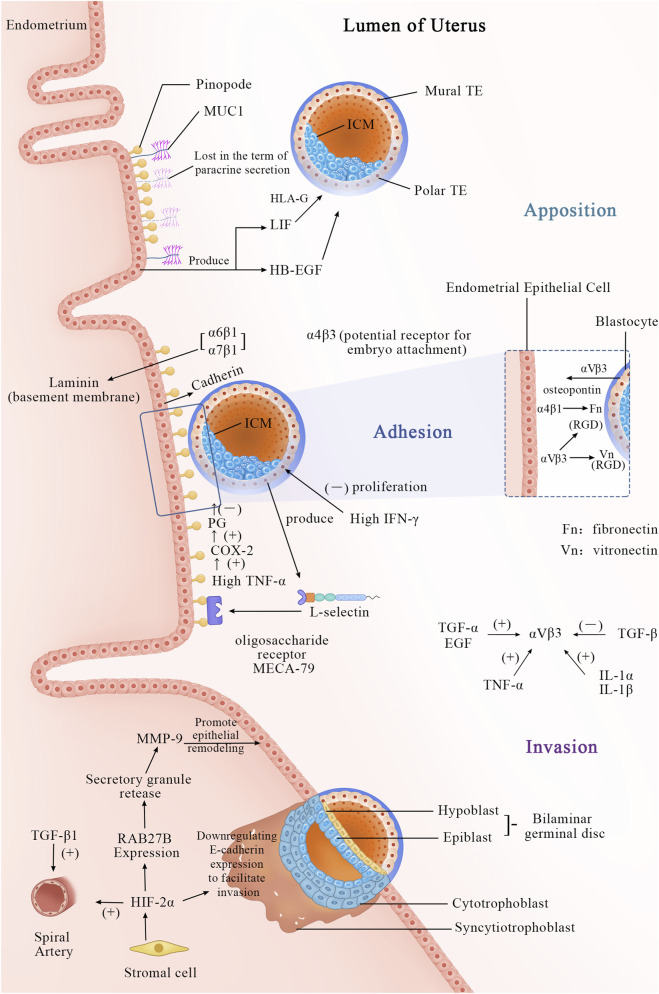
Apposition Phase (Day 5–6 Post-Fertilization): Cellular Events: The blastocyst completes hatching and is released into the uterine cavity. Through rotation and movement, the blastocyst makes initial contact with the uterine epithelial surface, where microvilli interact, but no firm adhesion is yet formed. The blastocyst adjusts its position and orientation, preparing for subsequent attachment. Molecular Events: A. The morphological feature of a receptive endometrium is the presence of apical protrusions on the luminal epithelial cells, described as “mushroom-shaped” or “sea anemone-like,” known as “pinopodes.” B. MUC1, a mucin, is expressed in the endometrium and may initially play a repulsive role to prevent non-implantation during the non-receptive period. At the end of the apposition phase, it is locally lost *via* paracrine secretion, assisting in positioning. C. Other molecules, such as LIF and HB-EGF, begin to participate as well. Adhesion phase (Day 6–7 post-fertilization): Cellular Events: The blastocyst establishes a stable, irreversible connection with the uterine epithelial surface *via* adhesion molecules. This stage anchors the blastocyst to the endometrium, preventing detachment. Molecular Events: A. MUC1 is completely cleaved, permitting direct attachment. B. Adhesion molecules, such as cadherins, integrins, and L-selectins, mediate intercellular connections. L-selectin is expressed on the surface of trophoblast cells and binds to L-selectin ligands (e.g., MECA-79) on the uterine epithelial surface, facilitating initial contact and orientation. C. The integrin subunit α4β3 is a hallmark of the implantation window. αVβ3 may serve as a potential receptor for embryo attachment, binding to the RGD sequence and initiating cell-cell interactions for trophoblast adhesion to the uterine epithelium. The αVβ3 dimer is present in both the uterine epithelium and trophoblast cells. This reciprocal interaction facilitates embryo anchorage: trophoblast αVβ3 recognizes uterine extracellular matrix (ECM) proteins, while uterine αVβ3 interacts with vitronectin and fibronectin expressed by trophoblast cells. The expression of αVβ3 is closely associated with several growth factors: TGF-α, EGF, TNF-α, IL-1α, and IL-1β, all of which can promote its expression under appropriate conditions, whereas TGF-β suppresses it. During embryo attachment to the endometrium, newly expressed integrin subunits α2, α6, and α7 indicate the blastocyst’s capability for implantation. Among these, integrins α6β1 and α7β1 bind to laminin, which is highly distributed in the basal membrane of the endometrial epithelium during the implantation window. Additionally, integrin α4β1, which is also expressed in the endometrium, interferes with fibronectin function in the trophoblast. D. During the adhesion process, the high expression of IFN-γ regulates the inflammatory microenvironment, decreasing the proliferation and invasion capacity of trophoblast cells. E. The increased secretion of TNF-α by the embryo is associated with implantation failure. TNF-α disrupts embryo-endometrium adhesion directly or indirectly by upregulating the COX-2/PGs pathway. Invasion phase (Day 7–12 post-fertilization): Cellular Events: The trophoblast differentiates into cytotrophoblast and syncytiotrophoblast. Trophoblast cells of the blastocyst invade the uterine epithelium, penetrate the stromal layer, and eventually embed deep into the uterine wall. This phase involves extracellular matrix degradation, vascular invasion, and decidualization, laying the foundation for placenta formation. Molecular Events: A. Stromal cells secrete HIF-2α, which reshapes the maternal epithelial barrier by activating the RAB27B-MMP-9 pathway, while also directly inhibiting E-cadherin to reduce cell adhesion. B. Meanwhile, TGF-β1 regulates the functional adaptation of the spiral arteries. The cytotrophoblast proliferates and differentiates into syncytiotrophoblasts, which, with the cooperation of these molecules, penetrate maternal tissues, completing embryo implantation.

Figure 1 the process of embryo implantation involves the initial interaction between an implantation-competent blastocyst and a receptive uterine endometrium, marking the first intimate dialogue between the two. This process, known as embryo implantation, is artificially divided into three stages: apposition, adhesion, and invasion (Enders and Schlafke, 1967). By the fourth day after ovulation, the blastocyst enters the uterine cavity through the tubal ostium and freely rolls within the uterine cavity. In mice, by the fourth or fifth day post-fertilization (pd4/pd5), the trophoblast differentiates into the polar trophectoderm (PTE), which contacts the inner cell mass, and the mural trophectoderm (MTE), which adheres to the uterine wall and initiates implantation (Bondarenko et al., 2023). Several factors, such as E-cadherin (Fleming et al., 2001), dynamic interactions between uterine epithelial cell actin cytoskeleton and integrins (Thie and Denker, 2002), facilitate the trophectoderm cells to adhere to the receptive uterine epithelial cells in appropriate positions and orientations. Subsequently, the invasive blastocyst penetrates the uterine epithelial cells, infiltrates into the stroma, and becomes enveloped by the uterine endometrium. Within the stroma, the embryo undergoes vascular wall remodeling and luminal widening (Pijnenborg et al., 2006) to accommodate high-speed blood flow (Carter et al., 2015), forming an extensive vascular network (Achache and Revel, 2006; Bhurke et al., 2020). This network supports embryo growth and ensures successful implantation.

The initial step of the implantation process relates to the interaction between L-selectin expressed on trophoblast cells and oligosaccharide ligands expressed on the endometrium. L-selectin, a cell adhesion molecule, plays a fundamental role during implantation (Heemann et al., 1994). Similar to its role in leukocyte-mediated early inflammation, L-selectin interacts with ligands on vascular endothelium, facilitating leukocyte rolling, adhesion, and migration. The rolling phenomenon of leukocytes parallels the attachment of blastocysts to the endometrial epithelium.

During invasion, trophoblasts, tasked with bridging the gap between placenta and uterus, are believed to utilize L-selectin to mediate interstitial migration (Prakobphol et al., 2006; Fazleabas and Kim, 2003). Studies indicate that L-selectin ligands such as MECA-79 and HECA-452 are upregulated during the implantation window. MECA-79 localizes to the endometrial epithelium throughout the menstrual cycle, aiding blastocysts in locating the optimal implantation site within the uterine cavity. Additionally, chemotactic and cytokine factors attract blastocysts to the optimal implantation site within the uterine cavity (Achache and Revel, 2006).

Most epithelial cells in the apex of the upper surface are protected by a glycocalyx, and endometrial epithelial cells are undoubtedly no exception (Aplin et al., 1994; Devine and McKenzie, 1992). Analysis shows that mucin MUC1 extends beyond the glycocalyx, potentially serving as the first molecule encountered by the blastocyst during implantation. MUC1 and MUC16 may have a negative influence on the implantation process, acting as an endometrial anti-adhesive molecule (Meseguer et al., 2001). During the receptive phase in humans, the endometrial epithelium is influenced by progesterone (Horne et al., 2006) and the interaction of the blastocyst (Meseguer et al., 2001), with MUC1 persistently elevated. When the blastocyst allows adhesion to the EEC (Endometrial Epithelial Cells) monolayer, MUC1 is locally lost only at the implantation site in a paracrine manner (Meseguer et al., 2001). The morphological characteristics of receptive endometrium include apical protrusions on luminal epithelial cells that can be described as “mushroom-like” or “sea-anemonae-like” (Nilsson, 1972; Psychoyos and Mandon, 1971). Originally termed “pinopods) (“drinking foot” in Greek) The term “uterodome” has also been used to dissociate these protrusions from an implied function. Most existing literature refers to these protrusions as pinopodes. Pinopodes are generally considered to be 5–10 μm cell protrusions at the apical surface of uterine epithelial cells, often associated with successful implantation (NILSSON, 1958; Usadi et al., 2003) and strongly regulated by ovarian steroid hormones (Quinn et al., 2020). Pinopodes serve as implantation sites, crucially providing an area unaffected by MUC1-mediated inhibition of embryo-endometrial interaction (Aplin, 1997). However, there remains controversy in the academic community regarding whether it is MUC1 or MUC16 that plays a role. Experimental evidence has detected the presence of MUC1 on pinopodes, while short interfering RNA (siRNA) knockdown of MUC1 does not affect the adhesion of trophoblast cells; conversely, in parallel experiments, knockdown of MUC16 using siRNA increases trophoblast cell adhesion (Gipson et al., 2008) Moreover, MUC1 has been implicated in potentially exerting significant influence on guiding blastocysts to appropriate implantation sites within the uterine cavity (Achache and Revel, 2006; Ashary et al., 2018). Simultaneously, MUC1 may facilitate blastocyst implantation; recent analyses have identified L-selectin on the surface of human blastocysts and complementary selectin ligands (such as sialylated oligosaccharides) on the receptive-phase uterine surface, supporting early blastocyst adhesion. However, it remains unclear which specific MUC1 molecules carry selectin ligands due to the glycan diversity of MUC1. MUC1 may participate in interactions with selectins, thus potentially facilitating blastocyst implantation (Carson et al., 2006).

Adhesion molecules such as integrins and calcium-binding proteins firmly attach the blastocyst to the implantation site of the endometrium to ensure successful implantation. The implantation process involves the blastocyst invading the superficial layers of the decidua, uterine myometrium, and spiral arteries of the uterus, while transitioning to an interstitial cell phenotype (Burrows et al., 1996; Vićovac and Aplin, 1996). This deep penetration of the uterine wall by trophoblast cells enables the placenta to securely affix itself and establish vascular connections between the two tissues (Merviel et al., 2001). Current exploration has identified various integrins present in the luminal and glandular epithelium of the human endometrium. During days 20–24 of the human menstrual cycle, three cycle-specific integrins—α1β1, α4β1, and αVβ3, as defined histologically—are co-expressed in the human endometrium, with only β3 mRNA expression showing an increase after day 20 (Merviel et al., 2001). The presence of both α1 and α4 subunits coincides with embryonic implantation during this window (days 20–24). Blockade of integrin β3 notably affects endometrial receptivity (Liu et al., 2013). The α4ß3 heterodimer serves as an excellent marker for the implantation window (Merviel et al., 2001), while αVβ3 may act as a potential receptor for embryo attachment. Experimental evidence has demonstrated the binding of αVβ3 to the RGD sequence, initiating cell-cell interactions necessary for attachment of trophoblast integrins to the uterine epithelium (Merviel et al., 2001). E-cadherin may possess a dual role in this process. Initially, its surface expression on cells is required for adhesion, whereas subsequently, E-cadherin may be downregulated to facilitate epithelial cell detachment and trophoblast invasion (Achache and Revel, 2006).

Research endeavor on the mechanism of embryo invasion into the endometrium is limited. Existing studies illustrate that during implantation, hypoxia-inducible factor 2α (HIF-2α), which is induced in stromal cells beneath the endometrium, plays a vital role early in pregnancy. HIF-2α is essential for the generation, survival, and/or maintenance of PGCs (Covello et al., 2006). As the embryo implants, rapid growth at the implantation site exceeds the blood supply, creating a relatively hypoxic environment (Covello et al., 2006). Under low oxygen conditions, hypoxia activates factors such as HIF-1α and HIF-2α. HIF-2α during implantation regulates the expression of RAB27B (Ma et al., 2022), a member of the Rab GTPase family that controls secretion granule release. These granules participate in the transport of MMP-9 from stroma to epithelium, promoting luminal epithelial remodeling during embryo invasion. Additionally, HIF-2α can directly or indirectly activate Oct-4 (Covello et al., 2006), which in turn activates Fgf-4, crucial pre-implantation growth factors necessary for embryo viability (Feldman et al., 1995). Evaluation also demonstrates that exposure of pre-implantation endometrial stromal cells to hypoxic conditions *in vitro* significantly enhances HIF2α expression. Thus, HIFs likely regulate the implantation process by modulating the uterine environment to a hypoxic state (Prakobphol et al., 2006). Furthermore, research project indicates that HIF-1α drives mouse embryonic stem cells to rely entirely on glycolysis for energy acquisition (Zhou et al., 2012). According to the study by (Maybin et al., 2018) et al., HIF-1α is essential for normal endometrial repair during menstruation, including genes associated with glucose metabolism (Wu et al., 2023) and angiogenesis (Meo and de Nigris, 2024). Due to the disappearance and remodeling of the spiral arteries during the menstrual cycle and pre-implantation endometrial preparation, the endometrium frequently experiences localized hypoxia and cellular oxidative stress (OS). This highlights the importance of the precise regulatory pattern of HIF-1α in the endometrium (Vaux et al., 2001; Gashaw et al., 2008). Under physiological conditions, the levels of HIF-1α fluctuate due to localized hypoxia, which is essential for tissue healing in the physiological regenerative process of endometrial receptivity preparation (Greenhill, 2018). This process has significant implications for endometrial decidualization (Dai et al., 2024).

### 3.2 The Warburg effect during embryo implantation

**FIGURE 2:**
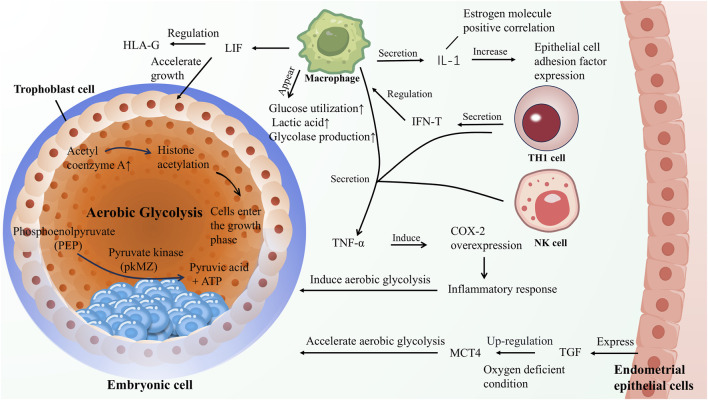
Within the embryonic cells, aerobic glycolysis is active, generating pyruvate and a limited yield of ATP enzymatically. Concurrently, acetyl-CoA facilitates the transition of embryonic cells into a growth phase by promoting histone acetylation. T helper 1 (Th1) cells modulate macrophage function through the secretion of interferon-gamma (IFN-γ), inducing alterations in the surrounding macrophages characterized by enhanced glucose uptake and increased expression of glycolytic enzymes. The interleukin-1 (IL-1) secreted by these cells, which shows a positive correlation with estrogen levels, upregulates the expression of adhesion factors in the endometrial epithelial cells, thereby promoting blastocyst implantation. Furthermore, leukemia inhibitory factor (LIF) secreted by macrophages stimulates the growth of embryonic trophoblast cells, accelerating embryonic development. Macrophages, Th1 cells, and natural killer (NK) cells collectively secrete tumor necrosis factor-alpha (TNF-α), which can induce aerobic glycolysis by provoking an inflammatory response. Simultaneously, the endometrial epithelial cells upregulate monocarboxylate transporter 4 (MCT4) under hypoxic conditions, further promoting aerobic glycolysis.

Figure 2 during the process of embryo implantation, physiological processes analogous to the Warburg effect may be observed. First, in early pregnancy, trophoblast cells proliferate rapidly and exhibit invasive properties, akin to cancer cells (Kohlrausch and Keefe, 2020). Secondly, embryos generate lactate-specific metabolism, creating a microenvironment at the implantation site characterized by high lactate levels and low pH (Gurner et al., 2022), similar to the microenvironment observed in the Warburg effect of cancer cells, where lactate is produced *via* glycolysis. Moreover, following implantation into the endometrium, embryos undergo angiogenesis within the stroma, remodeling blood vessel walls and widening lumens (Pijnenborg et al., 2006), to accommodate high-speed blood flow, thereby forming an extensive vascular network (Carter et al., 2015). Furthermore, studies reveal that during embryo implantation, trophectoderm cells undergo epithelial-mesenchymal transition to acquire appropriate adhesion and invasive abilities, which if uncontrolled, may contribute to cancer progression. This supports the notion of similarities between embryo implantation and the Warburg effect in cancer cells (Hantak et al., 2014). *In vitro* research proves that embryos appear to transition from oxidative to glycolytic metabolism pre-implantation, followed by a re-induction of oxidative metabolism post-implantation (Johnson et al., 2003). Thi T Truong et al. demonstrated that antioxidants can improve pre-implantation embryo development and viability in mice (Truong et al., 2016), further emphasizing the association between embryo implantation and the Warburg effect.

There is substantial glycolytic data supporting this hypothesis. The study by Jie Zheng (Zheng et al., 2023) et al. provides robust support for our hypothesis. In their investigation of HRT-FET cycles—that is, frozen embryo transfer (FET) performed during hormone replacement therapy (HRT) cycles—they observed a marked decrease in serum levels of TCA cycle substrates around the time of endometrial transformation. This suggests a potential reduction in oxidative metabolism *in vivo*, which closely resembles the metabolic features of the Warburg effect Weng et al. (2025). Found that impaired glycolysis can lead to defects in endometrial decidualization and contribute to infertility associated with endometriosis, indirectly indicating the importance of the Warburg effect for successful pregnancy. Most importantly, recent evidence clearly demonstrates that decidualization of human endometrial stromal cells is driven by Warburg-like glycolysis and regulated through mechanisms such as lactylation-mediated gene expression (Huang et al., 2025).

### 3.3 The role of mitochondrial dynamics in the process of embryo implantation

Mitochondrial dynamics play a crucial role in both blastocyst and endometrial tissue during the process of embryo implantation. Studies have shown that the balance between mitochondrial fusion and fission is essential for blastocyst formation, and disruption of this dynamic equilibrium significantly impairs blastocyst developmental quality, leading to abnormal cell lineage allocation, impaired energy metabolism, and the regulation of gene expression through epigenetic modifications (Sun et al., 2025). During the blastocyst stage, mitochondrial DNA (mtDNA) content significantly increases, and its replication, uncoupled from mitochondrial abundance, serves as a critical safeguard for overcoming developmental bottlenecks post-implantation (Winstanley et al., 2024; Shavit et al., 2025). Simultaneously, the functional status of the endometrial tissue directly influences implantation success, with premature senescence of endometrial stromal cells impairing decidualization through the accumulation of reactive oxygen species (ROS), thereby disrupting the interaction between the endometrium and the blastocyst (Deryabin et al., 2022). Changes in mitochondrial oxidative phosphorylation levels in endometrial stromal cells manifest as the downregulation of energy metabolism-related genes, such as PGC-1α, which may be associated with mitochondrial dysfunction in patients with repeated implantation failure (RIF) (Han et al., 2021; Han et al., 2023). These findings collectively reveal the pivotal role of mitochondrial dynamics in the coordinated interplay between blastocyst developmental potential and endometrial receptivity through the regulation of energy metabolism, gene expression, and intercellular communication.

## 4 How the Warburg effect affects endometrial receptivity

### 4.1 Immune modulation

Investigations imply that (Xu et al., 2021) nutrient metabolism and growth factor signaling are highly integrated processes. Glycolytic ATP serves as a variable resistor to measure the role of PI3K-AKT-FOXO1 signaling in T cell immune regulation, thereby elucidating as the Warburg effect. During implantation (Fujiwara et al., 2020), embryonic signals can induce immune tolerance and a robust local inflammatory response. The former prevents immune rejection reactions, while the latter promotes endometrial proliferation and maternal tissue remodeling crucial for embryo implantation and placental formation. It can be inferred that the immunomodulatory function of the Warburg effect is related to immune responses during embryo implantation.

Studies have shown that there exists a bidirectional regulatory relationship between energy metabolism and immune responses (Weerasinghe et al., 2024; Turner et al., 2016). In postpartum dairy cows, the defense of the uterine endometrium against bacterial infections primarily relies on innate immune mechanisms (Nash and Giles, 2025; Turner et al., 2012). However, when the organism is in a state of metabolic energy stress, this defensive capacity is significantly impaired, leading to reproductive disorders such as endometritis and infertility (Ji et al., 2025; Zhao et al., 2018). Specifically, inflammation significantly increases glucose consumption in the endometrium and induces metabolic reprogramming similar to the Warburg effect. This aberrant metabolic state may further exacerbate the decline in tissue glucose utilization. More importantly, energy metabolism stress significantly impacts uterine receptivity by interfering with glucose utilization and the AMPK signaling pathway (Zhang et al., 2022)—two core regulatory factors of cellular energy metabolism.

### 4.2 Microenvironment acidification

Embryos possess a unique metabolism that results in the establishment of a microenvironment characterized by elevated lactate levels and decreased pH (Gurner et al., 2022). Similarly, cancer exhibits aerobic glycolysis, thereby creating a comparable microenvironment (Apostolova and Pearce, 2022) to facilitate invasion of surrounding tissues.

Research indicates that lactate alters the endometrial receptivity remodeling process (Gurner et al., 2022), where lactate and reduced pH present in the microenvironment at implantation serve as early embryo signals to modify the function of endometrial epithelial cells, thereby enhancing endometrial receptivity and implantation initiation. Meanwhile, lactate and the associated decreased extracellular pH have been proven to convert the expression of several lactate-responsive genes and the epigenetic “lactylation” (Chen et al., 2021), further supporting this concept. During the establishment of uterine receptivity, the progesterone-dependent decidualization process significantly activates glycolysis and lactate synthesis. The produced lactate regulates redox homeostasis and apoptotic balance by inducing histone lactylation, a novel epigenetic modification, thereby ensuring successful embryo implantation. Inhibition of histone lactylation impairs the decidualization process (Zhao et al., 2023; Yang et al., 2022). Studies in ovine uterine tissue have revealed the crucial role of lactate in inducing H3K18 lactylation of the uterine endometrium and regulating redox balance and apoptotic equilibrium to ensure successful implantation. Concurrently, under pathological conditions, higher levels of lactate and lactate dehydrogenase A in ectopic endometrial tissue and ectopic endometrial stromal cells enhance histone H3 lysine 18 lactylation (H3K18lac), which can promote cell proliferation, migration, and invasion in endometriosis (Chen et al., 2023). This phenomenon was also observed in endometrial carcinoma cells, where histone lactylation was significantly elevated. Furthermore, histone lactylation regulates the expression of USP39, which, through interaction with PGK1, activates the PI3K/AKT/HIF-1α signaling pathway (Wei et al., 2024). Finally, the stimulation of glycolysis leads to the production of more lactate, further increasing histone lactylation. This reinforces the intimate connection between the Warburg effect in cancer and uterine receptivity.

Similarly, lactate can promote morphological and functional changes in other areas of the endometrium, manifested through modulation of stromal cell migration and promotion of endothelial vessel formation (Guido et al., 2012). Another aspect of lactate’s impact on endometrial receptivity remodeling, aside from changes in the luminal epithelium, is the differentiation of endometrial stromal cells, termed decidualization. Decidualization supports ongoing pregnancy through regulation of trophoblast invasion, support of vascular development, and formation of the placenta (Zuo et al., 2015), which are essential processes supported by maternal endometrial stromal cells for pregnancy (Lindsay et al., 2023). Lactate can trigger M2 or M1 macrophage polarization through oxidative phosphorylation and glycolytic regulation to exert decidual macrophage function (Gao et al., 2022). Warburg-like glycolysis and local lactate shuttle are activated during decidualization and play critical roles in supporting early pregnancy. Observations suggest (Young et al., 2014) that increased lactate levels may contribute to promoting the survival of ectopic endometrial cells and the establishment of lesions, akin to cancer cell metastasis. Endometriotic lesions and adjacent peritoneal tissues exhibit remarkably elevated glycolytic markers, suggesting metabolic phenotypic changes resembling the Warburg effect in tumorigenesis. Patients with endometriosis and cancer patients (Guido et al., 2012) are similarly influenced by TGF-β1 and demonstrate a significant lactate increase in peritoneal fluid (Young et al., 2014). This could potentially “feed” ectopic endometrial cells, enabling their survival, implantation, and invasion into the peritoneum, leading to endometriotic lesions.

### 4.3 Gene expression regulation

#### 4.3.1 Mechanism speculation

##### 4.3.1.1 Regulation of cell metabolism and growth genes

Recent research has demonstrated that the chromatin structure plays a crucial role in regulating various cellular functions, including DNA repair and gene transcription (Liberti and Locasale, 2016b). Furthermore, it has been found that glycolytic metabolism can influence chromatin structure (Liu et al., 2015). Now it is acknowledged that there is a direct link between cellular metabolism and the regulation of growth genes, with intracellular levels of acetyl-CoA potentially representing a widely conserved mechanism that promotes this important connection (Cai et al., 2011). It has been established that the substrate for histone acetylation, acetyl-CoA, can be regulated through glucose flux (Evertts et al., 2013). Elevated levels of acetyl-CoA may be sufficient to drive cells into a growth phase through histone acetylation (Lu and Thompson, 2012). Therefore, we hypothesize that the Warburg effect may regulate growth genes by altering cellular metabolism processes.

##### 4.3.1.2 The Warburg effect enhances glycolysis and glutamine breakdown to maintain cell proliferation

In normal functional cells, glucose is consumed *via* the tricarboxylic acid cycle to provide ATP for normal cellular physiological activities. However, in rapidly proliferating cells, aerobic glycolysis consumes large amounts of glucose to generate components essential for cell proliferation. The intersection between anabolic and catabolic pathways is primarily regulated by pyruvate kinase, which is re-expressed in its embryonic form as PKM2 (Mazurek et al., 2007). When PKM2 is inactive, it promotes anabolic metabolism (and branch pathways of glycolysis) (Lepleux et al., 2012), whereas active PKM2 catalyzes the conversion of phosphoenolpyruvate (PEP) to pyruvate, thereby generating one molecule of ATP.

Meanwhile, the inactivation of pyruvate dehydrogenase due to the Warburg effect blocks pyruvate from entering the tricarboxylic acid (TCA) cycle, forcing tumor cells to rely on glutamine as an alternative carbon and nitrogen source to sustain TCA cycle function (Delgir et al., 2021; Altman et al., 2016). This metabolic shift has been described as the “second Warburg effect,” where glutamine metabolism transitions from traditional catabolic pathways to nucleotide biosynthesis to meet the biosynthetic demands of malignant progression (Kodama and Nakayama, 2020). Mechanistically, the Warburg effect and glutamine catabolism are co-regulated in a synergistic manner: the increased glycolytic flux promotes lactate secretion through NAD+ consumption, while α-ketoglutarate (α-KG) produced from glutamine catabolism replenishes TCA cycle intermediates, together maintaining the acidic tumor microenvironment and providing biosynthetic precursors (Vallée et al., 2021; Chiarella et al., 2022). Computational biological models further confirm that the coupling of the Warburg effect with glutamine catabolism optimizes energy metabolism efficiency through linear programming, wherein glutamine not only serves as an energy substrate but also supports nucleotide synthesis *via* nitrogen metabolism reprogramming (Zhang and Boley, 2022; Ewald et al., 2024). Therapeutically, the glutamine metabolic reprogramming driven by the Warburg effect induces a “nutrient addiction” in tumor cells, providing a theoretical foundation for targeting the glycolysis-glutaminolysis axis as a therapeutic strategy (Lee and Kim, 2016). Recent studies also reveal that oncogenic pathways such as the Wnt signaling pathway can simultaneously activate both the Warburg effect and glutamine catabolism, further enhancing tumor metabolic plasticity through the promotion of macropinocytosis (Flores-Hernández et al., 2025).

#### 4.3.2 Genes and proteins may be involved

##### 4.3.2.1 MRAP2

Immunohistochemical evaluation revealed significant differences in MRAP2-positive staining among patients with idiopathic infertility. Compared to the control group, protein imprinting analysis of specific bands showed increased expression of MRAP2 protein in patients with idiopathic infertility (D'Aurora et al., 2019).

##### 4.3.2.2 BCL2L15

An animal experiment demonstrated upregulation of BCL2L15 in endometrial epithelial cells (EECs) of goats under treatment with progesterone (P4), estradiol (E2), and interferon-tau (IFN-τ). Yang et al. observed a prominent endometrial receptivity effect by specifically knocking down BCL2L15 using shRNA (Yang et al., 2020).

##### 4.3.2.3 PGC1α

As a central regulator of oxidative metabolism, PGC1α protects cells from oxidative damage by activating antioxidant genes while concurrently reversing the Warburg effect. During embryonic development, this metabolic regulation is critical for pre-implantation embryos in responding to oxidative stress, as the implantation stage necessitates a precise balance between aerobic glycolysis (Warburg effect) and oxidative phosphorylation to support rapid cell proliferation and differentiation (Lu, 2019).

##### 4.3.2.4 LDHA

This protein is a key enzyme in glycolysis, and its expression exhibits a decreasing trend from the cell stage to the blastocyst stage during embryonic development (Pu et al., 2020), which correlates with variations in the intensity of the Warburg effect. LDHA, as a hub gene in the gene co-expression network, significantly influences the gene expression pattern of embryos, and its mediation of lactate production plays a crucial role in regulating the acidic microenvironment during blastocyst formation and implantation (Li et al., 2025).

##### 4.3.2.5 ZC3H11A

Proteomic analysis reveals that this protein forms a close interactive network with mRNA export proteins in embryonic stem cells. Transcriptomic data confirm that the deletion of ZC3H11A leads to dysregulation of glycolysis and fatty acid metabolism pathways in embryos, directly affecting developmental competence at the blastocyst implantation stage. This indicates that ZC3H11A participates in the regulation of the implantation process by coordinating metabolic reprogramming (Younis et al., 2023).

##### 4.3.2.6 BCAT2

As a regulator of branched-chain amino acid metabolism, RNA-Seq data demonstrate that its expression during the blastocyst stage is influenced by the status of mitochondrial DNA. This protein, by linking amino acid metabolism to energy supply, may participate in regulating the intensity of the Warburg effect in pre-implantation embryos, while also impacting subsequent developmental potential (Tsai et al., 2018).

### 4.4 Regulation of cytokines

Endometrial receptivity refers to the comprehensive state that allows adhesion, invasion, and eventual implantation of the fertilized egg during the implantation process. This is regulated by various cytokines such as IL-1, IFN-γ, LIF, TNF-α, and TGF, among others. Abnormal endometrial receptivity or delayed establishment thereof can lead to implantation failure and subsequent infertility (Quinn et al., 2020).

Related studies highlight that cytokines such as IL-1, IFN-γ, LIF, TNF-α, and TGF (Guido et al., 2012) may promote glycolysis and the production of growth factors to participate in the Warburg effect. Macrophages, a functionally heterogeneous group of cells, are primarily formed by various microenvironmental stimuli. Zhu et al. propose that macrophages activated by interleukin-1β (IL-1β) and interferon-γ (IFN-γ) exhibit a Warburg effect similar to that in tumor cells (Zhu et al., 2015). Appelberg et al. recommend that IFN-γ activation of macrophages increases glucose uptake, enhances lactate production, and upregulates key glycolytic enzymes, thereby participating in the Warburg effect (Appelberg et al., 2015). Vaughan et al. propound that aerobic glycolysis in the Warburg effect can be induced directly by the inflammatory microenvironment (TNF-α), independent of additional genetic mutations and signals from adjacent cells (Vaughan et al., 2013). Jena et al. introduce to the public that TGF induces autophagy in cancer-associated fibroblasts under hypoxic conditions, promoting glycolysis in the Warburg effect through the upregulation of MCT4 (Jena et al., 2022).

#### 4.4.1 Interleukin-1(IL-1)

IL-1 is mainly derived from macrophages and can promote thymocyte proliferation. Hence it is referred to as lymphocyte activation factor (Dinarello, 1988). The IL-1 family consists of three members: IL-1α, IL-1β, and IL-1Ra. In recent years, the role of IL-1β in pregnancy has garnered increasing attention (Hantoushzadeh et al., 2020) and is believed to be associated with endometrial receptivity. Serum IL-1β levels are positively correlated with estrogen (Sunder and Lenton, 2000), and experimental evidence has shown that IL-1β levels are substantially elevated in patients with endometriosis primarily induced by estrogen (Montagna et al., 2008). Estrogen plays a key role in follicular growth and development as well as in endometrial growth. It can be inferred that IL-1β may participate in follicular growth, development, maturation, and induction of ovulation, potentially enhancing fertilization rates and embryo quality, thereby improving embryo implantation rates. IL-1 regulates endometrial receptivity by increasing the expression of epithelial cell adhesion molecules, thereby enhancing epithelial adherence to the blastocyst (Wang H. et al., 2018).

#### 4.4.2 Leukemia inhibitory factor (LIF)

LIF, a member of the IL-6 family, is a multifunctional cytokine with broad biological activities impacting various aspects of reproductive function (Onishi and Zandstra, 2015). These include the growth and development of oocytes, as well as the growth, differentiation, and implantation of embryos, making it a specific molecular marker for endometrial receptivity (Sharkey, 1998). LIF can promote the growth and proliferation of trophoblast cells, facilitating embryo adhesion and implantation. Additionally, it takes part in regulating trophoblast invasiveness by controlling the expression of human leukocyte antigen-G (HLA-G) in trophoblast cells (Chung et al., 2019). Therefore, increasing the expression levels of LIF in the endometrium may enhance endometrial receptivity and improve embryo implantation rates.

#### 4.4.3 Interferon-γ(IFN-γ)

IFN-γ is a hallmark cytokine of Th1 cells, capable of regulating the functions of other immune cells and promoting endometrial angiogenesis and vascular remodeling (Tayade et al., 2006), ensuring endothelial integrity (Jia and Li, 2020), thinning vascular walls, and increasing vascular permeability. Liu et al. (2017) present that elevated IFN-γ levels may induce excessive apoptosis of trophoblast outer layer cells, inhibit trophoblast cell line proliferation, hinder trophoblast invasion, and affect early embryo cytoskeletal formation. Zhang et al. (2015) proposed that IFN-γ can enhance the anti-apoptotic capability of *in situ* endometrial cells and stimulate cell adhesion molecule expression, thereby considerably influencing reduced endometrial receptivity (Qiu et al., 2020). Hence, excessive IFN-γ levels may decrease endometrial receptivity, exerting detrimental effects on pregnancy and embryo implantation.

#### 4.4.4 Tumor necrosis factor-α (TNF-α)

TNF-α is primarily produced by macrophages, Th1 cells, and NK cells, and it can stimulate the production of other pro-inflammatory cytokines (such as IL-1β and IL-6). It plays a paramount role during embryo implantation by promoting VEGF production, angiogenesis, and controlling trophoblast invasion, proliferation, and differentiation. Adequate TNF-α is necessary for maintaining pregnancy in women, as low concentrations can effectively defend against pathogenic microbial infections. While elevated levels of TNF-α can downregulate endothelial nitric oxide synthase (eNOS) and MMP-2 expression, promote prostaglandin production by inducing COX-2 overexpression, trigger inflammatory responses, and simultaneously inhibit trophoblast invasion, thereby hindering embryo implantation (Xu et al., 2017). Consequently, high levels of TNF-α can stimulate excessive proliferation of endometrial cells (Wang XM. et al., 2018) and reduce endometrial receptivity.

#### 4.4.5 Transforming growth factor -β (TGF -β)

TGF -β is a multifunctional peptide growth factor that stimulates proliferation of stromal cells, inhibits growth of epithelial-like cells, promotes extracellular matrix formation, suppresses immune function, and participates in angiogenesis. TGF-β1 exhibits differential expression in the endometrium at different stages: it is minimally expressed in the early and mid-proliferative phases in endometrial glands or stromal cells, whereas its expression notably increases in the late proliferative phase, predominantly in the stroma over glands. This suggests that TGF-β1 may promote endometrial growth *in vivo* and exert local estrogenic effects. TGF-β1 is minimally expressed during the early secretion phase but shows abundant cytoplasmic expression in glands and stromal cells during the mid-secretory phase, indicating its involvement in the formation of secretory endometrium (Erickson et al., 2004). TGF-β1 plays a role in both proliferative and secretory phases of endometrial development, promotes endometrial angiogenesis, enhances endometrial receptivity, and is one of the critical factors contributing to successful embryo implantation.

### 4.5 Hormone

#### 4.5.1 Estrogen

The placenta is the primary source of estrogen during pregnancy, particularly in humans and higher primates, where the biosynthesis of placental estrogen is distinctive. After the ninth week of gestation, the placenta gradually supplants the ovaries as the major organ for estrogen secretion, leading to a significant increase in circulating estrogen levels during the later stages of pregnancy (Albrecht and Pepe, 2010). Placental estrogen not only supports pregnancy by regulating progesterone biosynthesis and the maturation of the fetal hypothalamic-pituitary-adrenal axis, but also plays a pivotal role in the formation of the placental villous vasculature and the development of fetal ovarian follicles (Albrecht and Pepe, 2010).

In terms of endometrial receptivity, estrogen promotes decidualization and embryo implantation by optimizing the uterine microenvironment (Gellersen and Brosens, 2014; Lessey, 1998; Carson et al., 2000). Studies indicate that the transient estrogen surge occurring 7–10 days post-ovulation serves as a critical signal for the “window of implantation,” which regulates epithelial cell proliferation and stromal decidualization through the activation of estrogen receptor α (ERα) in the uterine epithelial and stromal cells (Simon et al., 2003; Pawar et al., 2015). Additionally, estrogen enhances uteroplacental blood flow and promotes angiogenesis (Reynolds and Redmer, 2001), thereby providing nutritional support for embryo implantation.

##### 4.5.1.1 Estrogen and metabolic reprogramming

Metabolomic analysis reveals that estrogen significantly affects the expression of genes associated with the mitochondrial electron transport chain, glycolysis, and the pentose phosphate pathway. This regulation may influence PARP-1 activity by altering the NAD/NADH ratio, thereby coordinating the relationship between the Warburg effect and estrogen synthesis (Riera Leal et al., 2020; Kaiser et al., 2020). *In vitro* experiments further confirm that estrogen exposure alters the metabolic phenotype of embryos, resulting in diminished mitochondrial respiration and increased lactate production in blastocysts generated *via in vitro* fertilization (IVF) (Lee et al., 2022; Chang et al., 2022). However, excessive estrogen stimulation may impair implantation potential by disrupting uterine receptivity (Liu et al., 2023). These findings reveal the dual role of the estrogen-Warburg effct axis in embryo implantation: moderate activation of Warburg effect benefits embryonic energy supply (Pu et al., 2020; Cagnone and Sirard, 2016).

##### 4.5.1.2 Estrogen receptors and their signaling mechanisms

The biological effects of estrogen are primarily mediated by two nuclear receptor isoforms (ERα and ERβ), which regulate gene expression in a tissue-specific manner (Arnal et al., 2017; Winuthayanon et al., 2014; Mata et al., 2015; Kuiper et al., 1996; Paech et al., 1997). ERα predominantly mediates the proliferative effects of estrogen in uterine epithelial cells, while the ERα signaling dialogue between epithelial and stromal cells regulates the process of decidualization (Winuthayanon et al., 2014). In contrast, ERβ is more involved in the relaxation of vascular smooth muscle, such as the ERβ in the mesenteric arteries during pregnancy, which mediates vasodilation to accommodate blood flow demands.

Recent studies have revealed that estrogen receptors not only exert their effects through the classical nuclear transcription mechanism but also trigger rapid non-genomic effects *via* membrane-initiated steroid signaling (MISS). For instance, ERα is localized to the cell membrane due to palmitoylation modification, and it rapidly regulates gene expression by activating kinase signaling pathways such as MAPK and PI3K (Arnal et al., 2017). This dual mechanism enables estrogen to coordinate short-term vasodilation with long-term vascular remodeling, as orphan nuclear receptors, synergistically enhance the expression of glycolytic genes in conjunction with HIF-1α during energy metabolism and angiogenesis (Cai et al., 2013), further expanding the complexity of estrogen signaling.

##### 4.5.1.3 Regulation of angiogenesis by estrogen

Angiogenesis is a critical process in endometrial receptivity and placental formation, involving endothelial cell proliferation, migration, and vascular maturation (Folkman, 1995). Estrogen directly promotes angiogenesis by upregulating the expression of vascular endothelial growth factor VEGF (Ferrara, 2004). For instance, during primate placental development, VEGF and its receptors drive the formation of the early pregnancy villous capillary bed by activating tyrosine kinase signaling (Reynolds and Redmer, 2001; Carmeliet and Jain, 2000). Estrogen also enhances uterine vascular permeability and endothelial cell proliferation (Johns et al., 1996), and promotes vascular smooth muscle cell hypertrophy through an ERα-dependent mechanism (Osol and Moore, 2014).

It is noteworthy that the angiogenic effects of estrogen are dual in nature (Laschke and Menger, 2016; Carmeliet, 2005). Under physiological conditions (such as pregnancy or endometrial repair), estrogen activates glycolytic enzyme PFKFB3 *via* G protein-coupled estrogen receptor (GPER1), promoting vascular regeneration in hypoxic tissues (Trenti et al., 2017; Prossnitz and Arterburn, 2015). However, in pathological conditions (such as endometriosis or tumors), estrogen-mediated aberrant angiogenesis may exacerbate inflammation and lesion progression (Laschke and Menger, 2016). For instance, tamoxifen, as a selective estrogen receptor modulator (SERM), can inhibit endothelial cell metabolic reprogramming and block tumor angiogenesis.

Additionally, ERRα, as a central regulator of energy metabolism, stimulates the expression of VEGF *via* a PGC-1α-dependent pathway, thereby promoting vascular repair following spinal cord injury (Hu et al., 2015; Puigserver and Spiegelman, 2003). This finding unveils the synergistic interaction between estrogen signaling and the energy metabolism network in angiogenesis.

Estrogen integrates the angiogenesis, energy metabolism, and cellular remodeling processes required for endometrial receptivity through a multi-receptor, multi-pathway mechanism (Simon et al., 2003; Cheng et al., 2023). The synergistic interaction between its classical nuclear receptors (ERα/ERβ) and membrane receptors (e.g., GPER1), along with the regulation of the metabolic-vascular network by estrogen-related receptors (ERRs), collectively ensures the establishment and maintenance of pregnancy (Cai et al., 2013). However, the complexity of estrogen signaling also leads to its dual role in pathological conditions, suggesting that future studies need to further elucidate its spatiotemporal-specific mechanisms in order to develop targeted therapeutic strategies (Laschke and Menger, 2016).

#### 4.5.2 Progestogen

Progesterone (PROG) is one of the earliest identified hormones, often referred to as a female steroid along with estradiol (Kolatorova et al., 2022). As a core hormone regulating female reproductive physiology, progesterone (P4) plays a pivotal role, particularly in the establishment of endometrial receptivity, embryo implantation, and the maintenance of pregnancy (Carson et al., 2000; Lessey et al., 1994).

##### 4.5.2.1 Progestogen and metabolic reprogramming

Progesterone plays a pivotal role in the process of embryo implantation, with its association to the Warburg effect primarily reflected in the regulation of metabolic reprogramming and embryonic development. Progesterone directly regulates endometrial receptivity through its receptor (PR) signaling pathway, for example, by downregulating miR-152 expression to inhibit GLUT3-mediated glucose uptake, thereby maintaining appropriate glucose concentrations in the uterine cavity to support embryonic development and implantation (Nie et al., 2019). Furthermore, progesterone enhances mitochondrial membrane potential in oocytes and early embryos, which may provide metabolic support for pre-implantation embryos (Dai et al., 2020). At the molecular level, the progesterone-PR signaling pathway modulates the deposition of hyaluronan (Hadas et al., 2020), cell cycle-related factors (such as IHH, BMP2), and key enzymes in glycolysis (HK2, PKM2, LDHA) (Chen et al., 2016), coordinating endometrial decidualization and embryo implantation. Notably, the intensity of the Warburg effect during embryo implantation dynamically changes, with glycolytic rates gradually decreasing from the morula to the blastocyst stage (Pu et al., 2020). Progesterone may ensure the synchronization of the embryo with the endometrium by regulating uterine receptivity (Hirota, 2019).

##### 4.5.2.2 Molecular basis of endometrial receptivity regulation

Progesterone induces the transformation of the endometrium from the proliferative phase to the secretory phase, establishing the “window of implantation (WOI)” (Gellersen and Brosens, 2014). Studies have shown that elevated levels of progesterone during the luteal phase significantly upregulate the expression of endometrial receptivity markers such as integrin αvβ3 and osteopontin (Carson et al., 2000; Lessey et al., 1994), providing a molecular foundation for embryo adhesion (Günther et al., 2023). Notably, local progesterone levels in the endometrium show no significant correlation with serum concentrations, which may be attributed to differences in the activity of steroid-metabolizing enzymes (e.g., 17β-hydroxysteroid dehydrogenase) within the endometrium (Huhtinen et al., 2012). Clinical research further confirms that in endometrial receptivity analysis (ERA™), the synergistic effect of local progesterone and 17α-hydroxyprogesterone has a statistically significant association with receptivity status (Labarta et al., 2021).

##### 4.5.2.3 Molecular synchronization and spatiotemporal regulation of embryo implantation

Embryo implantation consists of three stages: apposition, adhesion, and invasion of the blastocyst (Carson et al., 2000). Progesterone drives the proliferative-differentiation switch (PDS) by regulating molecular communication between endometrial epithelial and stromal cells (Gellersen and Brosens, 2014; Massimiani et al., 2019). For example, progesterone activates the Wnt/β-catenin pathway in stromal cells, promoting the secretion of leukemia inhibitory factor (LIF), which enhances the epithelial cell’s adhesion capacity to the embryo (Massimiani et al., 2019). Simultaneously, progesterone suppresses the expression of epithelial E-cadherin, reducing intercellular junction tightness and facilitating blastocyst invasion (Hirota, 2019). This spatiotemporal precision in regulation ensures the synchronization of endometrial and embryo development.

##### 4.5.2.4 Metabolic reprogramming supporting embryo implantation

Embryo implantation requires a substantial energy supply. Progesterone enhances glucose uptake and metabolic efficiency in the endometrium by upregulating the expression of glucose transporter 1 (GLUT1) (Zhang et al., 2020). Animal experiments have shown that progesterone treatment in mice significantly increases GLUT1 protein levels in the endometrium by 2–3 times, and glucose consumption rate correlates positively with the embryo implantation success rate (Zhang et al., 2020). This mechanism not only provides energy support for the embryo but also optimizes the local microenvironment’s pH balance through the regulation of metabolic byproducts like lactate, further promoting implantation.

##### 4.5.2.5 miRNA-mediated epigenetic regulation

Progesterone indirectly influences endometrial function by regulating the expression of microRNAs (miRNAs) (Shekibi et al., 2022). For example, progesterone upregulates the expression of miR-145 and miR-199 in human endometrial epithelial cells (HEECs), thereby inhibiting the expression of podocalyxin (PODXL). PODXL is a transmembrane protein that negatively regulates endometrial receptivity, and its downregulation significantly enhances embryo adhesion. *In vitro* experiments have shown that in endometrial cells transfected with miR-145 or miR-199, both the mRNA and protein levels of PODXL were reduced by 60% and 45%, respectively, and the embryo adhesion rate increased by more than 30% (Shekibi et al., 2022). This finding uncovers a novel pathway by which progesterone finely regulates endometrial function through epigenetic mechanisms.

##### 4.5.2.6 Dynamic balance between angiogenesis and decidualization

Angiogenesis is a crucial characteristic of endometrial receptivity (Gellersen and Brosens, 2014; Salmasi et al., 2021). Progesterone, in synergy with ovarian stimulation, significantly increases endometrial microvascular density by upregulating the expression of vascular endothelial growth factor (VEGF) (Zhang et al., 2020; Salmasi et al., 2021). Clinical studies have shown that exogenous progesterone treatment can elevate VEGF protein levels in the endometrium by 1.5 times and increase the density of CD31-positive endothelial cells by 40% (Salmasi et al., 2021). Furthermore, progesterone drives the transition of decidualization from the pro-inflammatory to the anti-inflammatory phase by secreting factors such as prolactin (PRL) and insulin-like growth factor binding protein 1 (IGFBP1), thereby limiting excessive trophoblast invasion and guiding its directional migration (Gellersen and Brosens, 2014). Abnormal decidualization, such as PR-A subtype defects, is closely associated with recurrent implantation failure (RIF) (Lydon et al., 1995).

##### 4.5.2.7 Diversity of receptor-mediated signal transduction

The functions of progesterone are mediated by its two receptor isoforms, PR-A and PR-B (Lydon et al., 1995). PR-A predominantly governs embryo implantation and pregnancy maintenance, with PR-A knockout mice exhibiting decidualization defects and implantation failure. In contrast, aberrant activation of PR-B leads to endometrial hyperplasia and chronic inflammation (Lydon et al., 1995). Moreover, the activity of PR is regulated by the estrogen receptor (ERα): estrogen during the proliferative phase induces PR expression through ERα, whereas progesterone during the luteal phase suppresses ERα *via* a negative feedback mechanism, preventing excessive endometrial proliferation (Chen et al., 2009). This dynamic balance between receptors is crucial for maintaining stable reproductive function.

##### 4.5.2.8 Synergistic regulation with estrogen

The synergistic interaction between progesterone and estrogen is essential throughout the reproductive cycle (Gellersen and Brosens, 2014). During the implantation window, progesterone promotes the differentiation of stromal cells and inhibits the proliferation of epithelial cells induced by estrogen, thus establishing a “proliferation-secretion” balance (Gellersen and Brosens, 2014). ERα knockout mice, which are unable to respond to estrogen signals, exhibit the absence of PR expression and arrested endometrial development (Chen et al., 2009). Clinical studies have confirmed that improper timing of progesterone supplementation during assisted reproductive technology (ART) may disrupt hormonal balance, leading to a shift in the receptive window and subsequent implantation failure (Paulson, 2011).

Progesterone precisely coordinates endometrial function through multiple mechanisms, including metabolic reprogramming, angiogenesis, epigenetic modifications, and receptor signaling pathways, thereby creating an optimal microenvironment for embryo implantation. Its effects are not only dependent on its concentration but also closely linked to the activity of receptor isoforms, local metabolic conditions, and synergistic hormonal interactions (Table 1).

**TABLE 1:** This table summarizes data related to the Warburg effect in both humans and mice during pregnancy.

Tissue/Model studied	Parameters measured	Observed changes	Proposed link to the Warburg effect during pregnancy
Human Endometrial Transformation Phase – Serum Metabolomics (Zheng et al., 2023)	Serum amino acids and TCA cycle intermediates	Levels of amino acids (e.g., ornithine, glutamate) are decreased. Levels of TCA cycle intermediates (e.g., malate) are decreased	The observed downregulation of metabolites reflecting TCA cycle activity in maternal serum during the window of endometrial receptivity suggests a metabolic shift in endometrial stromal cells—away from energy production and towards biosynthetic processes. This metabolic reorganization aligns with the features of the “Reverse Warburg Effect,” wherein stromal cells are thought to downregulate their own oxidative metabolism to generate a supportive microenvironment for embryo implantation.
Mouse Blastocysts (Lee et al., 2022)	Energy metabolism profile, lactate metabolism, intracellular pH	Enhanced glycolysis with suppressed oxidative phosphorylation; intracellular lactate accumulation and an increased lactate/pyruvate ratio	The metabolic pattern observed in these blastocysts is characteristic of the classic Warburg effect, demonstrating a clear preference for glycolysis and lactate production even under oxygen-sufficient conditions.
Mouse and Human Uterus (Endometrial Stromal Cells) (Huang et al., 2025)	Glucose uptake (e.g., GLUT1), key glycolytic enzymes (HK2, PKM2, LDHA), lactate production, extracellular acidification rate (ECAR), histone lactylation (H3K18la, H4K12la)	Markedly enhanced glucose uptake and glycolytic flux; increased lactate production leading to microenvironment acidification; upregulated expression of key glycolytic enzymes; elevated levels of histone lactylation.	Decidualizing cells exhibit metabolic characteristics typical of the Warburg effect. This metabolic reprogramming is understood to supply both the necessary energy and biosynthetic precursors required for cellular differentiation.

### 4.6 Age-dependent regulation

Existing research indicates that the effect of age on endometrial receptivity remains controversial. Some studies have found no significant differences in the expression of endometrial receptivity markers CD146 and PDGF-Rβ between the advanced age group and younger groups (p > 0.05), suggesting that patient age may not influence endometrial receptivity (Guo et al., 2023). Similarly, Abdalla et al. conducted a retrospective analysis of cases in which two different age groups of recipients shared oocytes from the same donor and received the same number of embryos. They evaluated the significance of recipient age (uterine age) on pregnancy rates, delivery rates, and miscarriage rates following egg donation, and concluded that the decline in fertility with age cannot be solely explained by uterine factors (Abdalla et al., 1997). However, other studies have reported significant decreases in the endometrial vascularization index (VI), flow index (FI), and vascular flow comprehensive index (VFI) in older women, along with markedly lower clinical pregnancy rates compared to younger women. These findings suggest that age may impact receptivity by altering the endometrial microenvironment (Wang L. et al., 2020). Furthermore, the prolonged duration of infertility associated with advanced age is significantly correlated with abnormal endometrial receptivity, manifesting as a higher tendency for older women to exhibit “early-receptive” or “pre-receptive” abnormalities (Opuchlik et al., 2025).

In assisted reproductive technology (ART), endometrial aging in older women may lead to a pro-inflammatory state and tissue fibrosis, and may alter the biological age of the endometrium through epigenetic regulation (Pathare et al., 2023). Research on the effect of age on the Warburg effect during the embryo implantation phase is currently lacking, highlighting the urgent need for further investigation by researchers in this area.

## 5 Current evidence gaps and potential future research directions

Due to the limited current evidence, further studies are required to validate this hypothesis. ① Lack of *in vivo* dynamic metabolic flux evidence: Current approaches, such as measuring lactate levels in culture medium or analyzing gene expression in tissues, cannot accurately determine the ratio of glycolysis to mitochondrial oxidative phosphorylation in endometrial energy metabolism during pregnancy. Consequently, it remains unclear whether the endometrium preferentially directs glucose toward lactate production *via* glycolysis or toward complete oxidation. Future research could employ isotope tracing techniques for metabolic flux analysis and fluxomics-driven determination of reaction free energy (Xu et al., 2020). ② Lack of functional validation of key metabolic enzymes and transporters: Molecules such as GLUT1, PFKFB3, and PKM2 have been highlighted in the literature as potentially critical in the metabolic process, but this assumption is based primarily on expression levels. Whether these molecules functionally contribute to establishing endometrial receptivity still needs verification. A promising future direction involves using CRISPR-Cas9 gene editing to knock out the corresponding genes, following the approach of Zou et al. (2025) in treating head and neck squamous cell carcinoma. Subsequent observation of glucose uptake, lactate production, and receptivity-related markers could then directly test if these nodal points drive receptivity. ③Lack of metabolic heterogeneity information at the endometrial cell subpopulation level: The endometrium is a complex system composed of epithelial, stromal, and immune cells, among others. Conventional bulk detection methods likely mask the unique metabolic patterns of distinct cell types. Therefore, during the window of implantation, it is unknown whether all epithelial cells undergo metabolic reprogramming or only specific subpopulations do. Single-cell RNA sequencing (scRNA-seq) could be applied to analyze the expression profiles of glycolytic pathway genes in individual cells, similar to how Chen et al. (2025) used scRNA-seq to identify transcription factors highly expressed in different cellular subpopulations.

By integrating these multi-level, complementary cutting-edge technologies, future research will be well-positioned to systematically elucidate the central role of the Warburg effect in endometrial receptivity. This work holds the potential to ultimately yield novel metabolic diagnostic biomarkers and therapeutic targets for infertility and recurrent pregnancy loss.

## 6 Conclusion and prospects

In summary, the processes of embryo implantation and the Warburg effect observed in cancer cells exhibit notable similarities. By integrating and analyzing clinical reports, connections can be identified across several dimensions: ① Immunomodulation (the inflammatory response induced by the uterus during the implantation process increases glucose consumption, thereby triggering the Warburg effect); ② Microenvironment alterations (the glycolytic activity of the blastocyst generates a high lactate and low pH environment, enhancing endometrial receptivity and angiogenesis); ③ Gene expression (glycolytic influences on genes such as MRAP2 and BCL2L15 may induce infertility); ④ Cytokine regulation (both cancer progression and embryo implantation demonstrate a requirement for and activation of multiple cytokines, including IL-1, IFN-γ, LIF, TNF-α, and TGF). Furthermore, during the implantation process, following the adhesion of the blastocyst to the uterus, trophoblast cells undergo epithelial-to-mesenchymal transition to acquire the necessary adhesive and invasive capabilities. If this process is uncontrolled, it may lead to carcinogenesis, indirectly highlighting the parallels between embryo implantation and the Warburg effect. However, current clinical and experimental research that clarifies the relationship between the two remains limited, and the underlying mechanisms await further exploration. This research could provide novel insights and directions for clinical studies and inform treatments for conditions associated with altered endometrial receptivity, such as infertility and recurrent miscarriage. Additionally, it presents a potential focus for traditional Chinese medicine approaches in treating infertility, representing significant research and translational implications. In the future, it may demonstrate remarkable efficacy in clinical settings, contributing profoundly to the field of maternal and child health globally.
