# Supplementary figures and images for: Linking the Warburg effect to endometrial receptivity: metabolic parallels in embryo implantation
Source: Front Cell Dev Biol. 2025 Nov 17;13:1683790. doi: 10.3389/fcell.2025.1683790 (PMC12665678; doi:10.3389/fcell.2025.1683790)

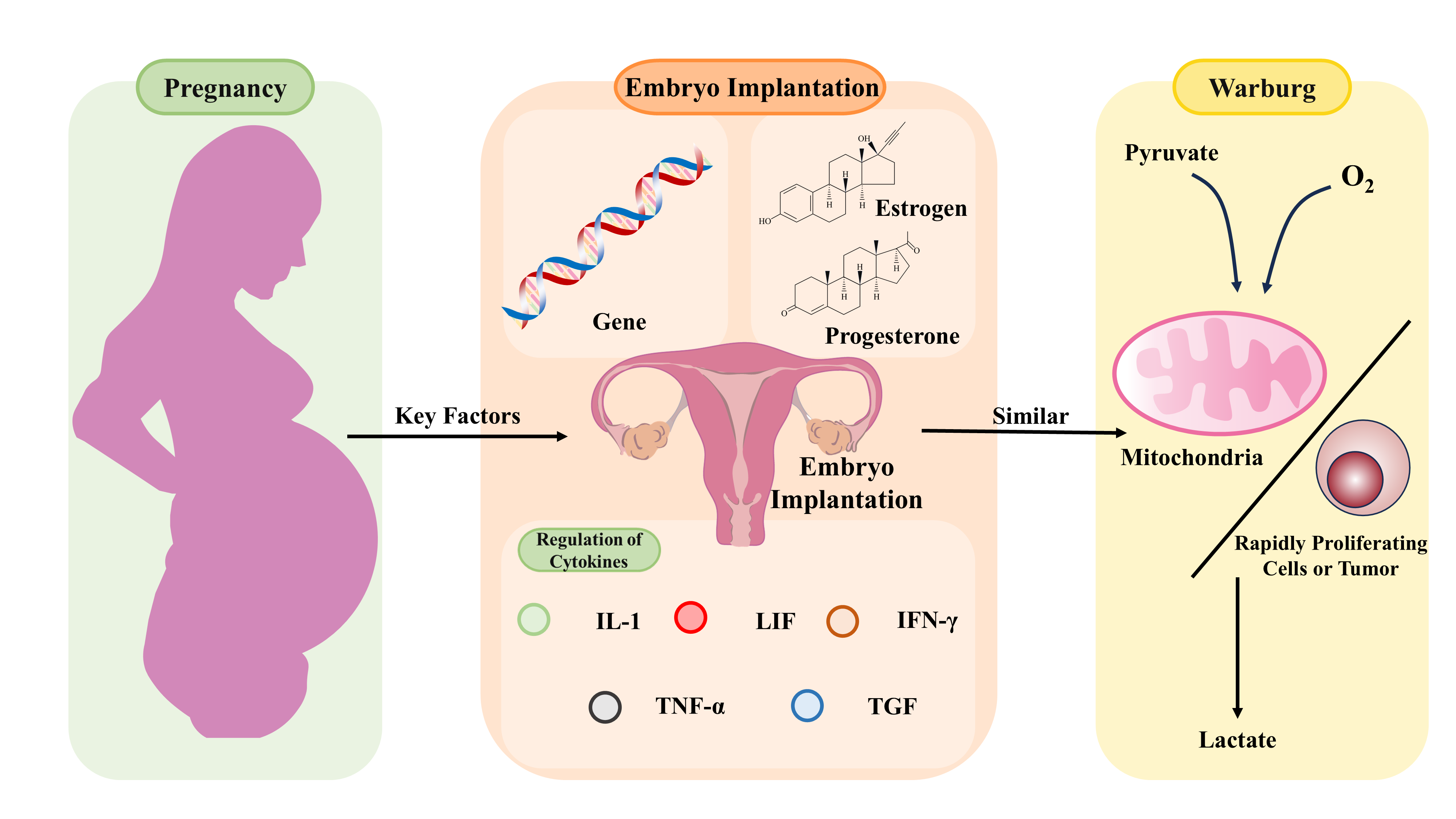

Supplement: Supplementary file 1 [file Image1.tif]
